# Exploring students’ learning experience in online education: analysis and improvement proposals based on the case of a Spanish open learning university

**DOI:** 10.1007/s11423-021-10045-0

**Published:** 2021-08-27

**Authors:** Pablo Rivera-Vargas, Terry Anderson, Cristina Alonso Cano

**Affiliations:** 1 Department of Teaching and Learning and Educational Organization, Universidad de Barcelona, Passeig de la Vall d’Hebron, 171. Edifici de Llevant, 2nd floor, 08035 Barcelona, Spain; 2 Facultad de Educación y Ciencias Sociales, Universidad Andrés Bello, Fernández Concha 700, Las Condes, Santiago, Región Metropolitana Chile; 3 Athabasca University, 10005 93 St. Edmonton, Athabasca, AB T5H1W6 Canada

**Keywords:** Online education, Autonomous learning, Digital divide, Competences in digital technologies, Student support

## Abstract

Not surprisingly, the number of online universities continues to expand—especially in Covid-19 times. These institutions all offer “online education” with diverse institutional, technological, and pedagogical processes. However, a fundamental element has to do with the experience of the students, and how they adapt to the educational model of the online university in which they are studying. In this article, we present the main results of the case-study developed in one of the most historical and relevant virtual universities in an international context. We have explored and analysed the process of adaptation to the educational model by the student body, and their perceptions of their interactions with the pedagogical, institutional, and technological elements designed to support their learning. Qualitative and quantitative methods are used to gather and analyse the data. From 1715 students who participated in the survey and the perceptions of 30 students individually interviewed, the results show positive evaluations regarding the integration and adoption of technological competencies, and also, that the online education generally serves as a responsive model to the emergent needs of the learner. However, the results also show that students have important concerns regarding the pedagogical and institutional support provided.

## Introduction

Online education has undergone profound transformations in recent times. Its evolution and configuration has gone hand in hand with changes that societies themselves have experienced with pervasive effects of the digital and networked society. These changes intensified during 2020 with the pandemic and its consequences.

The research presented in this article is from a case study developed at the Open University of Catalonia (UOC), located in Spain OUC was founded as an online university and has 25 years’ experience in offering online education. The main objective of the research was to explore and understand the academic and personal trajectories of the students during their educational experiences, with a focus on their interaction with the main pedagogical and technological elements that make up the online education system and their adaptation to the online education model used by the University. Considering this context, the two research questions that we answer in this article are:

- What are the most relevant aspects for the student body when evaluating their online educational experience?
- How can the educational experiences of virtual students in online universities be improved?

In order to answer these questions, and before presenting the methodological process carried out together with the results and main conclusions, two central dimensions of the work are analysed. First, the possibilities and limits of online learning, and second, a scan of the research literature on the nature and challenges faced by online students.

Although this research was carried out prior to the pandemic, we consider that its results are useful in identifying some of the possibilities and limitations that this form of education so as to improve the online educational experience of students from traditional, blended and online universities.

## Online education: possibilities and limits for learning

The definition of “Online Education” has gone through substantive transits. Although its definitions and approaches are varied, the present literature defines it as a mode that is essentially carried out in virtual learning environments (VLE), through the internet and with active use of digital devices (Anderson & Rivera-Vargas, 2020; Bates, 2019; Lee, 2019). Its emergence and consolidation must be understood not only as an evolution of traditional distance education, but also as a modality capable of dealing with the new formative demands of a technologically infused world (Lee, 2019) and a networked and connected society (Selwyn, 2019).

At the same time, the consolidation of the digital society, and the recent consequences and responses to the Covid-19 pandemic have reduced the distance between traditional face-to-face education and online education. Although their target audiences are still essentially different, traditional universities have undergone substantial virtualization processes, gradually leaving behind their analog heritage (Rivera-Vargas & Cobo Romaní, 2019). In addition, virtual universities have opted to offer, in their own virtual learning environments, some of those distinctive features of face-to-face education—including in some cases occasional face-to-face gatherings. An example of this is the continuing effort of virtual universities to strengthen the constructivist and collaborative character of their educational programming. Unlike early models of distance education that focussed on independent study (Fallon & Brown, 2016; Moore, 1989) and instructivist pedagogies (Zawacki-Richter & Anderson, 2014) online education now provides a platform and thus an educational teaching model with the affordances to create and sustain simultaneous and accessible learning communities anywhere at any time.

Online education has also defended its role as an inclusive educational modality that enables and facilitates access to higher education and the development of digital competences. Research efforts such as Sangrà et al (2012), and Hills (2017) show that the active use of electronic and digital media and devices in online education can facilitate access, development and improvement of the quality of education. Simultaneously to formal curriculum delivery, Chu (2010) and Anderson & Rivera-Vargas (2020) argue that online education provides and standardizes the technological and digital competencies of the students using the same virtual environment. This reduces the potential digital divide across multiple intersectional dimensions including gender, social class, physical disability, geographical location, and age (Chu, 2010).

It is important to highlight that these characteristics of online education, have coexisted for years with opposing critical views—some that question their effectiveness in real contexts rather than their global conceptualization. These highlights, for example, the limitations of mediated human contact and the need for the student to have high levels of personal motivation to be successful (Anderson & Rivera-Vargas, 2020; Kocdar et al., 2018). A problem also arises when the design of a virtual environment and learning activities is limited to the organization and dissemination of electronic resources (for example posting lectures online) and is not built on an ecological support of active learning (Davis et al., 2018).

In this sense, one of the most researched and most relevant aspects of the online education experience are efforts to increase student motivation in their educational process, through autonomy in learning, through effective use of digital tools, and through an active and interactive relationship between and amongst students and teachers. Guri-Rosenblit and Gros (2011) highlight the potential horizontal nature of this pedagogical relationship, giving relevance to the support that the student receives from the teaching staff and the institution as a whole. At the same time, Palloff & Pratt (2013), Kocdar et al., (2018) and Pilkington (2018) highlight the importance of helping students achieve autonomy and self-regulation so as, to motivate and thus enrich their educational experience.

In the next section, we delve more deeply into the importance of the student body in the educational model of online university education.

## The student in virtual learning environments

The development and large-scale accessibility to digital technologies and resources, together with the need for lifelong learning motivate why many people decide to pursue their university education in virtual environments (Jung, 2011; Pilkington, 2018). Many of these students cannot access traditional learning centres, with conventional face-to-face models, due to physical or economic constraints. However, they still need to acquire specific knowledge and skills that are applicable to their personal and professional lives (McKnight et al., 2016). In addition, it is usually students with professional experience and digital skills, who generally seek an education that allows them to integrate their previous knowledge, with new skills and knowledge (Jung, 2011; Sánchez-Gelabert et al., 2020) while adapting to their emergent needs in their professional and personal lives.

From a constructivist viewpoint, Anderson (2016) and Vuopala et al (2016) maintain that the learning process with digital tools is or at least can be, fundamentally collaborative. Students create knowledge through interaction between themselves, the teacher, and their environment, that allows and indeed forces them to assume the leading role in their learning process. The demographic characteristics of the student body engaged in online learning are heterogeneous. Jung (2011) and Murphy and Stewart (2017) noted that the majority of the first wave of online education students made contact with the computer and with digital technologies in late youth or adult life (late twentieth century and early twenty-first century). That is, these students came from a campus-based educational environment where the teacher was the leader of the process, who set the timetable and dictated how knowledge would be acquired.

The following generations of virtual students are made up of a great variety of ages, the majority coming from a regulated formation focused on the transmission of knowledge made by the teacher, but who more prone to proactivity (Murphy & Stewart, 2017). Thus, they are more accustomed to collaboration between equals, to be more democratic, more diverse and be involved in less hierarchical telematic relationships. Although there are differences and varying needs among online students according to their culture, the disciplines they choose to study and their age, they show many common characteristics in their identity and performance when learning in these environments (Kocdar et al., 2018; McKnight et al., 2016). Perhaps the most striking commonality, although not surprising, is that the majority enrol for the first time in online education, without knowing what it is to be an online student, without knowing what to do, what it entails, how to perform optimally and without having received any training (Jung, 2011; Pilkington, 2018). Despite this, most are able to adapt and learn due to the flexible context, transference of digital skills from social and professional contexts, having the individual responsibility for their time and the ability to access educational resources until the completion of their course (Pilkington, 2018).

When referring to the online student, there is a tendency to highlight those actions that describe their predisposition to participate in online learning environments (Sánchez-Gelabert et al., 2020). This is important if we take into account the multiple transformations that have occurred in the educational field during the last decades (Lee, 2019). In fact, the figure of the student in virtual environments as an apprentice with a higher level of autonomy, not only emerges as a consequence of the development and use of digital technologies in educational contexts, but also emerges from previous efforts aimed at positioning the student as a leading actor in the teaching and learning processes, and as a result strengthens their autonomous learning (Farrell & Brunton, 2020). Palloff & Pratt (2013), for example, suggest the profile of the successful online student, that although emergent and mediated through the use of computers, is marked by the abilities and skills to manage the tools and resources of these learning environments. The student also gains skills and competencies that facilitate their autonomous learning. Palloff & Pratt (2013) identify these characteristics of the online students:

- Ease of sharing their work, points of view, and experiences with others in order to build virtual communities.
- Improvement in written communication skills, in order to relate to others online and to develop social capital by exposing personal and communication skills.
- Ability to self-motivate and self-discipline, given the flexibility of the courses.
- Commitment to the course, investing a significant amount of time and effort.
- Adaptability—a critical position in their learning process.
- Understanding that reflection is part of the learning process and that learning is a transformative experience.

The use of digital technologies in the teaching and learning process aims to partially remedy the deficiencies of the traditional teaching and learning model used on campus and formally used in older distance education models (Alqurashi, 2019). In this way, inserting strategies that encourage the student to work autonomously, reinforce their self-control and support leaving behind the conception of student as passive, dependent, rigid, solitary, and non-reflective are critically important (Murphy & Stewart, 2017; Sánchez-Gelabert et al., 2020).

## Method and context

### Context of the Open University of Catalonia

This case study focuses on the Open University of Catalonia (UOC). This institution was founded in 1995 during the period of the initial internet expansion. The UOC sought to be an academic environment adapted to the characteristics of modern society (UOC, 2009). It is recognized as one of the first universities in the world that has supported its teaching and learning model with the integral use of a virtual learning environment (VLE) or a learning management system (LMS) (Grau-Valldosera & Minguillón, 2014).

During the 2019–2020 academic year, the UOC had 56500 active students, of which 40500 were undertaking bachelor’s degrees studies, and 16000 were undertaking master’s degrees studies. From its foundation (1995) until December 2020, there have been a total of 89300 Bachelor’s degrees, and master’s degrees graduates,^1^ spread over 134 countries around the world (UOC, 2020).

According to the institutional report for the 2018–2019 academic year (UOC, 2020), the typical student who begins studies at the UOC combines studies with work (81.70%), works in the private sector (67.40%), is studying to progress professionally (61.10%) and opts for the UOC in order to reconcile studies, work, and other responsibilities (50.40%) (UOC, 2020). Finally, it should be noted that the UOC has been increasing its international prestige in recent years. The latest version of the university ranking created by the Times Higher Education journal (THE, 2021), has placed the UOC in the following quality dimensions: among the top 150 young universities; the second-best Spanish university under 50 years old; the best online university in Ibero-America; and among the top 601–800 global universities.^2^

#### Approach to the educational model

The UOC's educational model is focused on extending the learning possibilities of the student. To this end, it offers a wide diversity of strategies, resources, and pedagogical work dynamics, based on support of the teaching team and on interaction with classmates, who try to empathize with their needs and lifestyles (UOC, 2020). The model supports students learning as they work and communicate on the network (Sánchez-Gelabert et al, 2020).

The UOC’s educational model, it is based on the integration of four fundamental elements (Grau-Valldosera & Minguillón, 2014; UOC, 2009, 2020):

- The commitment to a horizontal and collaborative relationship between students and teachers.
- The promotion of student autonomy and self-regulation, placing these at the center of their learning process.
- Human support of the student, instantiated by three roles:
- “Consultant”: providing pedagogical and subject matter support on a course-by-course basis
- “Tutor”: providing institutional pastoral support throughout the students’ enrolment^3^
- “Technological support department”: providing technical support and accompaniment
- Resources and content (spaces, tools and didactic materials with active use of digital technologies).

In addition, the UOC supports its pedagogical commitment with evaluative flexibility and the creation of a learning environment that favours interactivity and cooperation between students, and between students and the university (Sánchez-Gelabert et al., 2020). It is a model focused on accommodating the contemporary students as active participants in their learning processes (Kocdar et al., 2018).^4^

### Methodological design

In this research, a sequential mixed methods approach (qualitative first) has been used, complementing the use of qualitative methods with quantitative information collection and analysis techniques (Goetz & LeCompte, 1988). At the analytical level, an interpretative view is assumed, because it emphasizes the concern for the local and for the generation of a knowledge that is relevant and that emerges within these environments. This has also allowed us to observe how the students from an online university interact with educational transformations, and what realities and subjects they recognize (Anderson & Rivera-Vargas, 2020). The study focuses on how reality is generated through ordinary actions. We explore how students, beyond the technology itself, create and recreate learning contexts from their interaction with digital technologies (Laux et al., 2016). Thus, the research can be considered a multi-method, single case study (Stake, 1995) bounded by undergraduate students enrolled in the Open University of Catalonia (Spain).

### Information collection tool

Individual active interviews were conducted (Denzin, 2001; Holstein & Gubrium, 2020) with students, academics and institutional representatives of the UOC. In this type of active interview, the interaction between the interviewer and the interviewee tends to be symmetrical, that is, both having an active role. We used semi-structured interviews with topics and questions derived directly from the objectives of the research (Denzin, 2001), but also left room for participants to expand and shape the conversations. Table 1 shows the main topics developed in the interview.

**Table 1:** Main topics of the semi-structured interview guideline

No.	Main topics
1.	Previous experience in university studies
2.	Previous knowledge about online education
3.	Motivations to do online university studies
4.	Professional expectations regarding the studies carried out at the UOC
5.	Main personal characteristics of the UOC student body
6.	Personal organization to carry out online university studies
7.	Relationship between and with other students
8.	Development and assimilation of new digital skills while studying at the UOC
9.	Personal assessment of the students on the virtual learning environment used by the UOC
10.	Personal assessment of the relationship and communication between the student body and the UOC
11.	Personal assessment of the UOC's educational model
12.	Personal assessment of the studies carried out at the UOC

At the quantitative level, based on the statements and discourses obtained in the analysis of the interviews, a questionnaire was designed (Goetz & LeCompte, 1988). These complements and triangulate the information obtained in the qualitative phase, and at the same time provide a more representative reflection of the experiences of those interviewed. To ensure the content validity of the questionnaire, the initial 40 questions were validated through expert judgment. Seven experts from the field of online university education and distance education participated, from Spain (3), Canada (2), UK (1) and Chile (1). This process was carried out through validation matrices, where each expert responded individually with a Yes or No to the validity conditions of each question. Of the 40 questions, 35 obtained a quality assessment of over 75%, while 5 questions that were evaluated under 75%, sere dropped in the final evaluation instrument. Thus, the final instrument was made up of 35 questions, which included closed multiple-choice responses, and Likert scales.^5^

The internal consistency of the instrument was calculated and interpreted using data from a test application. The results, α = 0.60, indicated a «good» internal reliability for scales between 0.6 and 0.8 points (Cohen et al., 2007). The test application process that led to the final design of the questionnaire spanned two months. The data from questionnaire were downloaded and saved in an IBM SPSS 25.0 (2017) spreadsheet with consideration for the ethical aspects corresponding to anonymity and data security compliance. The questionnaire was administrated using the online platform of the UOC Office of Planning and Quality.

### Sample and participants

The research targetted students in undergraduate degree programmes, with current enrolment at the time of the research (course 2016–2017), and who have had at least two consecutive years of experience at the UOC. The process was divided into two phases. In the first, essentially qualitative phase three undergraduate degree programmes were reviewed these were: computer engineering, psychology, and business administration and management (BAM). This choice was not arbitrary, since these three studies are the oldest (Computer Engineering) and with the highest number of students (Psychology, and BAM) (UOC, 2009, 2020). In addition, each of these undergraduate degree programmes belong to a different knowledge area (Table 1). Finally, 30 individual active interviews of students across the three selected degree programmes were carried out (10 students from each). These students were selected based on three criteria:

- Had completed at least two full years at the UOC
- Have passed all the subjects enrolled
- Voluntarily to participate in this investigation. An invitation was sent to all students who met the first two criteria. Each of the 30 interviewees responded with an email confirming their personal interest in participating in this investigation.

In the second, quantitative phase, the complementary questionnaire (Goetz & LeCompte, 1988) was provided to all 7885 students belonging to the 15 undergraduate studies that made up the UOC’s enrolment in 2016. The questionnaire was completed by 1715 (21.75%) of students (which fits a 3% accepted error and a confidence level of 99%).

### Analysis procedure

The analysis of the qualitative information used discourse analysis by grouping and categorizing the responses from the interviewees. We selected this type of analysis from Wetherell & Potter (1998), because it poses discourse as a social practice, and not just as a set of statements. In the words of Iñiguez & Antaki (1994) we extracted “a set of linguistic practices that maintain and promote certain social relationships” (1994: 63).

In the qualitative information coding and treatment phase, the transcripts were grouped according to the three-degree studies considered (computer engineering, psychology, and BAM). Then, the codified process was developed from the interview guideline. Subsequently, the units of meaning created in each degree were grouped into a single frame of group narrations. This work reduced the volume of data, highlighting those collective narratives directly and indirectly linked with the research objectives. By systematically reading the codes, selected citations and their context, we searched for patterns, themes and regularities, as well as contrasts, paradoxes and irregularities (Denzin, 2001; Denzin et al., 2016). From this, we proceeded to relate the codes, grouping and regrouping them until they made sense in order to create consolidated discourses. The regrouping of narratives generated a new analytical sense, allowing in this way, new interpretative schemes (Wetherell & Potter, 1998). This work gave rise to four categories:

- Assimilation of digital competences
- Flexibility and adaptation to the UOC model
- Virtual learning environments
- Students’ support

Once the categories were organized, they were analysed according to a combined model, in which the content of the narratives was worked on, also considering their discursive form, recovering analytical resources from the repertoire model of Wetherell & Potter (1998).

For the analysis of the quantitative information, the data was analysed using SPSS software, using descriptive analysis techniques. The classification and tabulation was made according to the Knowledge Areas in which the UOC organizes the degree studies, shown in Table 2.

**Table 2:** Relation/-knowledge areas and bachelor’s degrees

Knowledge areas	Bachelor’s degrees	No
Arts and humanities studies	Humanities Catalan language and literature	202
Studies in information sciences of communication	Communication information and documentation	150
Law and political science studies	Criminology law labour relations	250
Economics and business studies	business management marketing and market research tourism	339
IT, multimedia and telecommunications studies	Computer engineering, multimedia, Telecommunication technologies	173
Studies in psychology and education sciences	Social education psychology	601
Total		1715

Finally, in the last phase, analytical triangulation and discussion was carried out using both the qualitative and quantitative information obtained. The coherence and correlation between both types of information was analyzed, identifying the most significant similarities and differences.

## Analysis of results

The results presented below have been organized based on the development of the four emerging categories mentioned in the previous section. Each of the four categories were addressed using triangulation of data, including the constructed discourse, quotations from the interviewed student body, and descriptive results of the supplied questionnaire, illustrated in tables, that have been grouped according to the Knowledge Areas described in Table 1.

### Assimilation of digital competences

One of the main results of this research was to determine if the online education experience itself provided powerful and sustainable digital skills to students. This is significant because it was not a goal established in the pedagogical models proposed by UOC (UOC, 2009, 2020), nor in the initial expectations of this research.

An important part of the student body considers this process of assimilation of skills in digital technologies as an added value to the formative experience, as affirmed by this fifty-two-year-old student of Psychology:

You are using the virtual campus, then an application, and then you have to create one of these, etc. At first it is difficult to understand the mechanics of them, but once you manage to do it for the first time, everything flows and is faster (Psychology student).

Although a significant majority of the students stated that they had some level of digital competence prior to joining the UOC, almost all of the participants interviewed acknowledged that they now use digital technologies more actively and reflectively in other areas of their work and leisure activities. This they attribute to their formative experience using e-Learning tools.

**Fig. 1:**
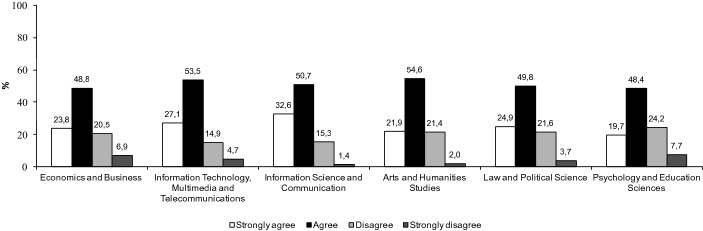
I consider that studying at the UOC using online education technologies has allowed me to gain new technological competences. According to discipline (%)

Results obtained from the questionnaire, confirm results gathered from individual interviews. As we see in Fig. 1, students from all areas of knowledge acquired new technological skills.

**Fig. 2:**
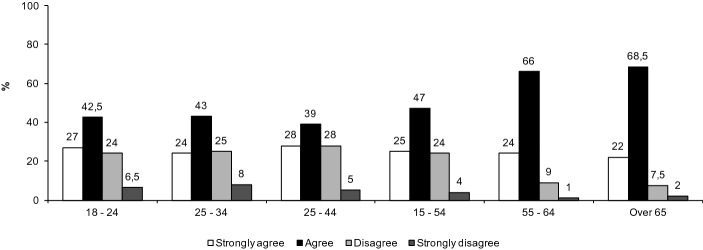
I consider that studying at the UOC under the online education modality has allowed me to assimilate new technological competences. According to Age (%)

As might be expected, we see a trend (Fig. 2) towards more perceived value of online education skills acquired in older ages. Beyond this, the evidence shows that age differentiates students when it comes to assimilating competences in digital technologies during the online education experience.

Complementing this data, we found from students’ comments, that variables such as: experience with digital technologies, gender, age, degree of study, do not negatively affect the academic interaction between classmates, nor their learning performance in VLE. This is in line with Chu’s (2010) argument that online education experience may reduce the digital divide between ages, genders and disciplines.

However, the main obstacle to interaction with colleagues is more related to the lack of concrete experience in the virtual campus of the UOC, as this student of BAM states.

You can quickly find out who is harder to study with online. If you are a new student, everything is slower (when you have to work in a group), they also fill the forum with questions, send you some personal messages, etc. The key is in the years (level of experience) you have been in the UOC, if you have several, then you do everything right. This goes beyond the age or sex of the people, or other aspects (BAM Student).

Thus, the increased participation in VLEs, in addition to favouring the assimilation of new digital skills, is valued by the student body as an action that tends to reduce the digital divide between students.

### Flexibility and adaptation to the model

In this second dimension, students’ evaluations of the online education educational model proposed by the UOC are revealed. At the same time, the compatibility of the model with their own lifestyles is analysed. The flexibility of the learning model (Sangrà et al., 2012), together with the largely asynchronous character (Jung, 2011), allowed for continuing studies while engaged in often busy professional and personal lives. According to Anderson (2016), Anderson & Rivera-Vargas (2020) this generates greater commitment and leadership of the student in their own learning process:

Studying from my house and doing other activities at the time of day that suits me best, is fundamental for me. Otherwise, (using a face-to-face model) I couldn’t work or have a personal life (Psychology Student).

Because of my work, I have a free schedule at very unusual times. That’s when I can take advantage of studying and doing evaluative activities. Having this autonomy and leading my learning process, is what, in fact, allows me to be committed to my studies and motivates me to continue (Computer Engineering Student).

Complementing this discourse, the results of the questionnaire reaffirm that students across the UOC's Knowledge Areas consider that the online education educational model at the UOC allows them to combine their studies with the usual organization of their time, and also, with their personal and professional life (Table 3).

**Table 3:** Studying under the online education modality has allowed me to combine my professional and academic life without major inconvenience (%)

Knowledge areas	Strongly agree	Agree	Disagree	Strongly disagree
Economy and business	84.2	14.9	0.6	0.3
IT, Multimedia and telecommunications	84.2	15.8	0.0	0.0
Information and communication sciences	83.8	16.2	0.0	0.0
Arts and humanities	83.2	15.2	0.5	1.0
Law and political science	78.6	20.6	0.4	0.4
Psychology and education sciences	82.0	16.6	0.5	0.8

The results also reflect that many virtual students of the UOC opt for online education because their lifestyle prevents them from attending university institutions with face-to-face instructional models:

In order to get a job promotion, for me it was important to finish my studies, but studying in person was impossible. So, I looked for distance education alternatives and the truth is that it works very well for me. So far, I have not had to change my routine in any way (Computer Engineering Student).

**Fig. 3:**
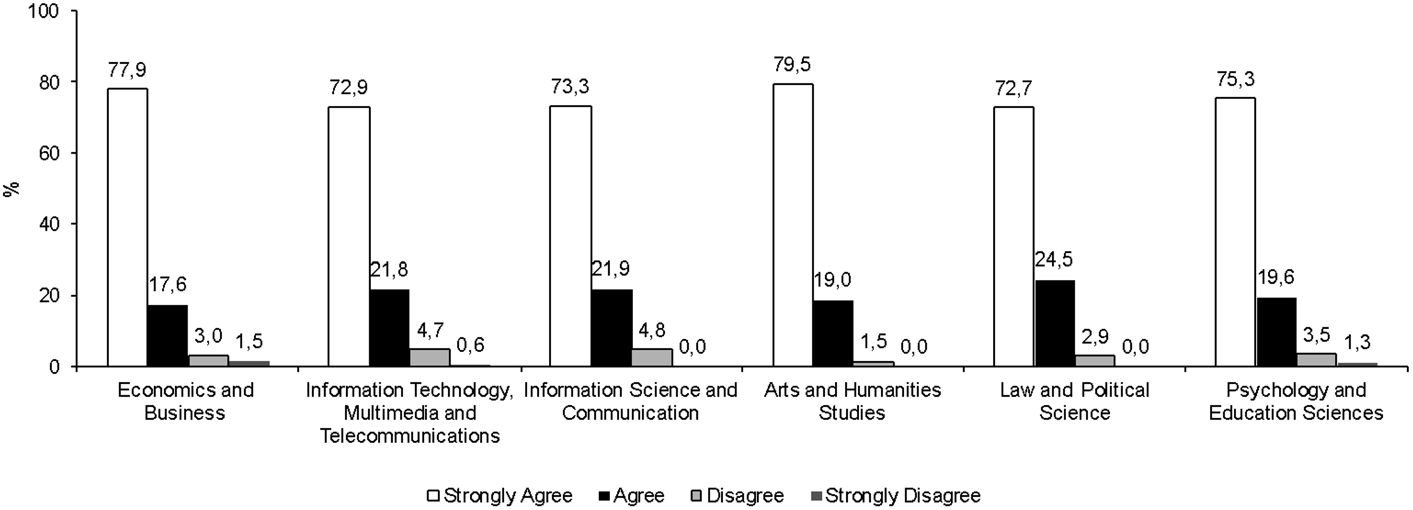
With the lifestyle I have, it was very difficult for me to study in person. (%)

As can be seen in Fig. 3, this sentiment is felt beyond the field of study or knowledge areas.

**Fig. 4:**
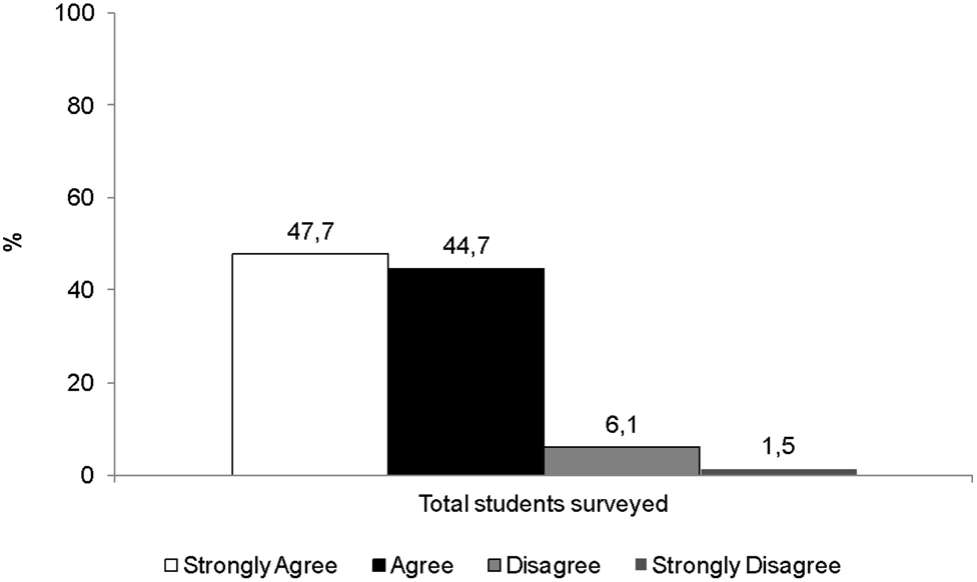
If the continuous assessment model did not exist (through the CET), I could not study at the UOC (%)

One of the central aspects of the UOC's educational model is its permanent evaluation component, based on Continuous Evaluation Tests (CET). This flexible evaluative model, which aims to promote the autonomy and leadership of students in their educational process (Sánchez-Gelabert et al., 2020; UOC, 2020), represents the main reason why students choose to complete their university education in the online education system (Fig. 4).

However, beyond the fact that the student recognizes the flexibility of the UOC's educational model, there are aspects such as “personal academic organization and planning” that are recognized as difficult challenges to solve. This is significant, given the importance of enhancing the self-regulation and autonomy of students throughout their educational process (Pilkington, 2018; UOC, 2020). In this sense, beyond the flexibility of the evaluation model (considering evaluation and pedagogical tools), students also find that due to other commitments of their lifestyle, it is often difficult for them to plan and fulfil their academic responsibilities.

The model is flexible, and it is assumed that you can self-manage everything, but in practice, it does not stop having a very intense lifestyle. I finish each semester overwhelmed and pushing my academic abilities to the limit (BAM Student).

In general, we found that students organize and submit their evaluation work without sufficient time, and without much space for reflection and content review. In the opinion of the interviewees themselves, this has as a consequence for many, that, it is not possible to complete the courses with high levels of academic quality.

### Virtual learning environments

In this third section, the perceptions and the assessment of the structure and design of the virtual campus, are presented. The student perception of the virtual campus, where most student interaction action takes place, is generally positive. It is recognized that it is a friendly environment and that the applications and tools provide an effective learning space. One student explains:

Without having much previous experience in these environments (VLE), the truth is that I have always found that the (virtual) campus of the UOC is easy to manage. It is quite instinctive. In addition, the fact that you can give it your own design, makes it more representative of your own identity (Psychology Student).

Most students had positive opinions of the design, technical performance and operations of the virtual campus, however we found (Table 4) that the assessments of the students tend to vary depending on the area of knowledge and the variable that is evaluated. For example, students in the knowledge areas: law and political sciences (7.94), and Arts and Humanities (8.16), give a very good general assessment of the virtual campus. While for the students of the knowledge areas: computer science, multimedia and telecommunications (7.67), and Information Sciences (7.38), the assessment tends to be less favourable. Table 4 also shows that generally students generally rate positively access to materials, assessment activities, and communications with consultants, tutors, student services and peers. There are however small differences between knowledge areas.

**Table 4:** Virtual campus assessment by dimensions

	Economics and Business Studies	IT, Multimedia and Telecommunications Studies	Studies in Information Sciences of Communication	Arts and Humanities Studies	Law and Political Science Studies	Studies in Psychology and Education Sciences	General note by dimension
Access to the pedagogical materials of the subjects	8	7.71	7.66	8.18	8.1	7.93	**7.95**
Access to Continuous Evaluation Tests (CET)	8.15	7.61	7.64	8.22	8.03	8.04	**8**
Virtual tools to contact "consultants" and "tutors"	7.86	7.68	7.76	8.3	7.89	7.81	**7.88**
Virtual tools to contact the institutional areas (student service, technical service, etc.)	7.2	7.01	6.95	7.58	7.42	7.08	**7.19**
Virtual tools to contact other students	7.36	7.06	7.18	7.81	7.37	7.34	**7.36**
Personal email	7.95	7.61	7.65	8.07	7.86	7.9	**7.87**
General note by Knowledge area	**7.84**	**7.67**	**7.38**	**8.16**	**7.94**	**7.73**	**7.8**

Average rating (from 1 to 10) according to knowledge area

Generally, we note that the two aspects with worse valued are related to the communication mechanisms of the campus, both for communication with institutional departments (7.19), and communication between students (7.36). The two most valued aspects of the virtual campus are: access to pedagogical materials of the subjects (7.95) and access to continuous evaluation tests (8).

### Student support

A successful student's support from UOC staff is probably one of the most important aspect of UOC's educational model (Sánchez-Gelabert et al, 2020). Both the interview and survey results show that although a small majority of students report satisfaction with tutors and consultants, there is a large portion of students who express dissatisfaction with these support roles (see Table 5). When we are asking about the feedback that students receive from the Consultants, the interviewees value it as weak and inefficient, recognizing how frustrating this is.

In general, you spend a lot of time doing your work, so you expect relatively clear feedback. But nothing, just a note, you do not know the reasons behind it. It seems to me very insincere (BAM Student).

Corrections should be returned so that you can see how you have done in your work and what criteria were used to evaluate them, because if not, in the end, the only thing you look at is whether you have passed or not (Psychology Student).

**Table 5:** Overall assessment of the performance of the tutor and the consultant

Knowledge areas UOC	Valoration	Consultant (%)	Tutor (%)
Economics and business studies	Very good	16.9	30
	Good	40	21.5
	Bad	37.9	44.2
	Very bad	5.2	4.3
IIT, multimedia and telecommunications studies	Very good	22	32.9
	Good	39	23
	Bad	33.3	39.9
	Very bad	5.7	4.2
Studies in information sciences of communication	Very good	19.3	23.6
	Good	36	28.5
	Bad	40.7	41.9
	Very bad	4	6
Arts and humanities studies	Very good	41	45
	Good	40	30
	Bad	13.8	19
	Very bad	5.2	6
Law and political science studies	Very good	26.3	25
	Good	22.7	22
	Bad	49.2	40.4
	Very bad	1.8	12.6
Studies in psychology and education sciences	Very good	23	25
	Good	26.5	20.7
	Bad	47.3	44.8
	Very bad	3.2	9.5

In relation to the performance of Tutors, many participating students consider that their efforts are not very resolute when it comes to managing and solving certain problems:

In the three years that I have been at the UOC, I have only managed to contact my tutor 2 times and I will have written more than 20 emails. It is very frustrating, although I confess that at least it is good to know that they exist, that, in case of emergency, you can still count on them. In fact, if this role did not exist, surely people would ask for it (BAM Student).

In any virtual environment, the supportive role of human contacts remains critical to student success (Tait, 2014). As seen in Table 5 this area of support is problematic for many students as revealed in the questionnaire. For example, if the four indicators of the Likert scale are grouped in only two: “positive” (“Good” and “Very good”) and “negative” (“Bad” and “Very bad”), we observe that except for “Arts and Humanities Studies” the results in general, are equally split between positive and negative evaluations.

As we see in Table 4, dimensions such as technological management, adaptation to the educational model and flexibility of its evaluation model, present generally positive evaluations from the students. However, when assessing two of the most relevant actors in the process of student's accompaniment and support, as shown in Table 5, the results are not so positive. The only exception is given in the area of “Arts and Humanities Studies”, where the assessment of the performance of tutors and consultants is substantively positive. Meanwhile, in the five remaining areas of knowledge, there is a symmetry between the sum of the indicators “Very good—Good” and “Very bad—Bad”, highlighting even, the cases of the areas of “Law and Political Science” and “Studies in Psychology and Educational Sciences” where the sum of the negative evaluations exceeds 50% of the total, both in the case of Consultants and Tutors.

For me, the tutor’s performance has been very bad. At first, I did not know how the virtual campus worked, I did not know what and how many subjects I had to do. I did not even know if it made much sense to do college at my age. I wrote several emails, asking for your guidance. He never answered me (Psychology Student).

This is lack of communication was also identified by students from other areas of knowledge, where although the opinion on the performance of the consultants and tutors is better, we have been able to recognize multiple manifestations that openly criticize their performance.

Since I entered the UOC, I had to do everything by myself. I have never felt that a consultant oriented me well pedagogically, even his feedback tends to be monosyllabic. From the tutor, I have nothing to say, in three years, I still do not know who he is (BAM Student).

This data shows that the pedagogical support to the student, which represents one of the most significant aspects of online education (Almusharraf & Khahro, 2020; Sánchez-Gelabert et al., 2020; UOC, 2020) is not satisfactory for large numbers of students. These assessments of the role of the “Consultants” and “Tutors” reveal some significant weaknesses in the UOC’s educational model.

## Discussion and conclusions

In this section, we will answer the two questions that guided the research.

Regarding the first question: “*From the perception of the students of the Open University of Catalonia, Identify and analyse which are the most relevant aspects for the student body when evaluating their online educational experience*?” The development of the empirical phase of this research has allowed us to identify four categories that have been relevant to students during their online university education. Next, we will contrast the evidence that supports these categories with the literature on the subject that we presented in the first part of this article.

### Assimilation of digital competences

We find that participation in virtual environments had a significant side effect of providing opportunity to learn and assimilate digital competences (Hills, 2017). Thus, participation in a virtual environment has benefits that go beyond subject matter learning objectives. Further, these benefits have become critical for both personal and professional advancement as communications and networking applications become increasingly important in both commercial and social realms. As Anderson & Rivera-Vargas (2020) argue, these benefits are seen across all age groups suggesting generally positive outcomes.

### Flexibility and adaptation to the model

This outcome highlights that the students carry out their university education through online education because it gives them the possibility of making their studies compatible with the normal development of their professional and personal lives (Guri-Rosenblit & Gros, 2011; Pilkington, 2018). This critical capacity of increasing access is well known in the literature and widely supported among students of different ages in this study. We find that one of the major reasons why students opt for online education is due to the enhanced flexibility of the model as compared to attendance in traditional university institutions with a face-to-face approach (Anderson & Rivera-Vargas 2020). In relation to the mechanisms of evaluation of learning, students prefer the continuous evaluations tests (CET) to learge summative examinations, because is suits their lifestyle (allowing for more flexible time-shifting. However, similar to expressed in Kocdar et al (2018) the student’s question being overworked in certain subjects leading to lack of time for reflection and application of new knowledge.

### The virtual campus

In general terms, and similar to the evidence that Sánchez-Gelabert et al. (2020) presented, students made positive evaluations of their experience of the technological platform and technical support, especially in those aspects of essentially educational including: access to pedagogical materials and to Continuous Evaluation Tests. However, contrary to what Fallon and Brown (2016) suggested, the lowest scores were obtained in those dimensions that evaluated the virtual contact tools between the students with the University (at a pedagogical, institutional, and technical level) and among the students themselves.

### The student’s support

One of the most central aspects in determining the success of the online educational experience is the quality of interactions with human staff (Palloff & Pratt, 2013; Pilkington, 2018; Anderson & Rivera-Vargas 2020). In the case of the UOC, pedagogical and institutional accompaniment to the student body is supposed to continue throughout their educational experience, whereas it is absent or provided on a course by course basis in most other open universities (Davis et al., 2018). In this way, this university gives responsibility to both tutors and consultants, to provide student support (UOC, 2009). UOC contends that this has been authentic and sustainable model (intact since 2010) (UOC, 2020). However, the results show that there are medium to high levels of dissatisfaction with these resources as assessed by the students. This is an important factor, considering the efforts made by this educational institution to promote the students' interactive experience during their university studies. There are strong pedagogical rationale documenting the value of this type of continuous support (Sánchez-Gelabert et al., 2020), but it seems at UOC that the realization of this value is at best uncertain. This study reveals the need to first measure and then design ways to make this support more effective. This diagnosis should be accentuated when evaluating the performance of tutors and consultants.

Regarding the second question: “*From the analysis of the results of this research: how to improve the educational experience of virtual students in online universities*?”

As we have seen, the educational model of the UOC works well in general and has a good evaluation by the student body. However, there are some aspects that require adjustments or at least a revision. Based on this, and bearing in mind the results of this study, some initiatives are proposed below that could be considered by online universities to improve the educational experience of their students.

Firstly, the assimilation of digital skills during their own online education experience is an aspect recognized and well valued by the student body. These are instrumental skills, which are necessary to make adequate use of the virtual campus. However, at different moments of the research, the students raised the need for their educational experience to further strengthen the comprehensive and reflective processes. Given this, the main suggestion in this dimension is to go beyond an instrumental learning and promote the assimilation of reflective and critical skills of the student body on the use of digital technologies. For example, it would be useful to make students literate about the potential of the use of data in education, incorporating in the educational process the possibility of working and generating new knowledge (both individually and in groups). Self-care strategies could also be strengthened in relation to learning to properly organize the time they spend exposed and connected to digital screens. It could be useful in this sense to propose the development of learning activities outside the virtual campus, or directly in communities that are not virtual nor online.

Secondly, although the evaluation proposal based on CET is one of the strengths of the UOC’s educational model, an important part of the student body claims the need to reduce the time they dedicate to performing the multiple tasks that it entails. Given this, a commitment to the project-based learning model (PBL), could fit in and solve this demand of the student body to have more time for reflection and the application of the new knowledge assimilated in a subject. There is already a set of PBL, DIY, Maker experiences, implemented with substantive successes in other online university institutions (Lasauskiene and Rauduvaite, 2015; Chu et al., 2017). In most cases, these are initiatives that, through projects, have connected the study plan of the subjects with real social problems of interest to the students. It is a strategy that, in addition to being flexible, makes it possible to strengthen the bond of the student body with their own learning trajectory (Miño-Puigcercós et al., 2019).

Thirdly, the results show the need to generate alternative strategies, that favor the accompaniment of the student, taking into account their diverse learning needs and socio-cultural characteristics (Tait, 2014). However, the support from the human actors—tutors and consultants present a significant number of students expressing dissatisfaction with the service levels. At the same time, provision of this service incurs both financial and opportunity costs in addition to time commitments required of both students and staff. Thus, choice of type and level of support is critical. A first step in decision making is to accurately measure the current function of the service within the delivery system (Oregon et al., 2018).

A second step, given the current post-pandemic scenario is to look at innovative models from other industries in which service is provided by machines (for example driver-less cars, chat bot customer service, self-service banking and automated tax returns, etc.). This growing set of digital tools used effectively by online universities may provide pedagogical, institutional, and technical support to students (chatbots, web tools, student access to their own and comparative student data analytics), which according to Salmon (2013), could favour the experience of the student body in virtual learning environments in addition to extending their expertise and exposure to digital technologies.

Finally, the study provides useful information for UOC, but also to other virtual universities and traditional face-to-face mode universities that are experimenting important changes given the accelerated virtualization caused by the Covid-19 pandemic (Dhawan, 2020). The results also can assist the wider online educational community by examining, in detail, an innovative model for online learning. Thus, each institution can reflect on their own level of support and service to students and costs of doing so.

## Data Availability

We will make the data available.

## Footnotes

1 See more details here: https://www.uoc.edu/portal/en/metodologia-online-qualitat/fets-xifres/index.html.

2 See more details here: https://www.timeshighereducation.com/world-university-rankings/open-university-catalonia.

3 This role is relatively unique amongst online universities in that the term ‘tutor’ is often used elsewhere to refer to faculty who engage only with the student as they are enrolled in a particular course (similar to consultant role above), not as here, where they are engaged with the student throughout their enrolment at the university (Sánchez-Gelabert et al., 2020).

4 For more information on the UOC's educational model see https://www.uoc.edu/portal/en/metodologia-online-qualitat/model-educatiu/index.html.

5 See the final version of the questionnaire, before it was digitized and included in the UOC’s institutional platform, and sent to the student body: https://drive.google.com/file/d/1frnPPJsiTBULI92RQgx1jI4bcaAQB4GN/view?usp=sharing.
